# Cardiosome mediated regulation of MMP9 in diabetic heart: role of mir29b and mir455 in exercise

**DOI:** 10.1111/jcmm.12589

**Published:** 2015-03-30

**Authors:** Pankaj Chaturvedi, Anuradha Kalani, Ilza Medina, Anastasia Familtseva, Suresh C Tyagi

**Affiliations:** Department of Physiology and Biophysics, School of Medicine, University of LouisvilleLouisville, KY, USA

**Keywords:** cardiosomes, exosomes, exercise, matrix metalloproteases, diabetes, microRNAs

## Abstract

‘Cardiosomes’ (exosomes from cardiomyocytes) have recently emerged as nanovesicles (30–100 nm) released in the cardiosphere by myocytes and cardiac progenitor cells, though their role in diabetes remains elusive. Diabetic cardiovascular complications are unequivocally benefitted from exercise; however, the molecular mechanisms need exploration. This novel study is based on our observation that exercise brings down the levels of activated (Matrix Metalloprotease 9) in db/db mice in a model of type 2 diabetes. We hypothesize that exosomes that are released during exercise contain microRNAs (mir455, mir29b, mir323-5p and mir466) that bind to the 3′ region of MMP9 and downregulate its expression, hence mitigating the deleterious downstream effects of MMP9, which causes extracellular matrix remodeling. First, we confirmed the presence of exosomes in the heart tissue and serum by electron microscopy and flow cytometry, respectively, in the four treatment groups: (*i*) db/control, (*ii*) db/control+exercise, (*iii*) db/db and (*iv*) db/db+exercise. Use of exosomal markers CD81, Flottilin 1, and acetylcholinesterase activity in the isolated exosomes confirmed enhanced exosomal release in the exercise group. The microRNAs isolated from the exosomes contained mir455, mir29b, mir323-5p and mir466 as quantified by qRTPCR, however, mir29b and mir455 showed highest upregulation. We performed 2D zymography which revealed significantly lowered activity of MMP9 in the db/db exercise group as compared to non-exercise group. The immunohistochemical analysis further confirmed the downregulated expression of MMP9 after exercise. Since MMP9 is involved in matrix degradation and leads to fibrosis and myocyte uncoupling, the present study provides a strong evidence how exercise can mitigate these conditions in diabetic patients.

## Introduction

Exercise has been described as a polypill 1 and it mitigates diabetic complications. Exercise can also diminish cardiac dysfunction in diabetic patients but the molecular mechanisms still remain unclear. Exercise brings down the levels of MMP9 in aorta 2 though the molecular events are yet to be explored. We have reported earlier that genetic deletion of MMP9 improves the contractile function in cardiomyocytes 3 and improves cardiac repair in the heart of diabetic patients 4. In this study, we hypothesize that exercise can bring down the levels of MMP9 by releasing exosomes from the muscles and contain paracrine factors like microRNAs which can silence MMP9.

In an earlier report we showed that exosomes contain molecular conservatories from their cells of origin and mitigate endothelial cell dysfunction 5. Exosomes are small membrane vesicles (40–100 nm) and contain paracrine factors like microRNAs and help in cell to cell communication 6. Exosomes have been reported to be released in the cardiosphere by telocytes which are interstitial cells with long projections 7,8. Extracellular vesicles from cardiac progenitor cells have been reported to inhibit apoptosis of cardiomyocytes and improve heart function after myocardial infarction 9. Exosomes are critical agents of cardiac repair and are potential candidates for therapy 10. Although many studies have reported role of exosomes in cardiac repair 11 and myocardial remodelling 12 but the mechanism how exosomes exert their beneficial effect is unknown. Therefore, this study explaining that exercise releases exosomes that contain microRNA to silence MMP9 can bridge a major gap between exercise, exosome mediated beneficial effects to heart and diabetes. Diabetes being a major challenge and affecting 8% of the U.S. population 13, will affect 400 million people worldwide by 2030 14 with prevalent cardiovascular deaths. Diabetic complications mitigated by exosomes (released during exercise) can delineate novel mechanisms for coping up with cardiac fibrosis and uncoupling. Exosomes contain circulating microRNAs which can modify the expression of genes 15,16 and we screened mir466, 455, 323 and 29b that can bind to the 3′ UTR region of MMP9 and downregulate its expression. In type 2 diabetic rats, one of the factors that are responsible for anti-angiogenesis is exosomes released by cardiomyocytes that carry mir302 to the endothelial cells 17 and hence, affect the gene expression in the target cell.

Although myocytes have been reported to release exosomes *in vitro* 18 but studies are lacking to show that exercise releases exosomes. Since exercise brings down the levels of MMP9 2 it can mitigate the fibrosis and myocyte uncoupling that are caused by the activated levels of MMP9 19,20. In addition, our previous study suggests that in diabetic patients MMP9 causes matrix remodelling 21 and ablation of MMP9 induces survival and differentiation of cardiac stem cells in the heart of diabetic patients 4. Hence, inhibition of MMP9 has been proposed as a therapeutic approach to treat cardiovascular dysfunction 22 by using chemical agents 23 or drugs but this has not been successful so far. Our hypothesis that exercise releases exosomes containing microRNA to silence MMP9 can pave the way to novel therapeutic strategies.

## Material and methods

### Animal models and experimental groups

To study the role of exercise in diabetes we are using db/db mouse as models of type 2 diabetes. We have selected four experimental groups: (*i*) db/+ control, (*ii*) db/+control +exercise, (*iii*) db/db and (*iv*) db/db+exercise (mice procured from Jackson Laboratory, Sacramento, CA, USA). The db/+ control and db/db group of mice were exercised on a treadmill (Exer 3/6 Treadmill#1, Columbus, OH, USA) with a controlled speed (7 m/min. for db/db mice and 10 m/min. for db/+ controls) for 300 m/day, 5 days/week 2. For adaptation of mice to exercise, the treadmill speed was kept at 7 m/min. on the first day, and then was increased by 1 min. each day until the maximum speed of 10 m/min. at the end of the second week; after that, the speed was kept constant. For db/db mice the speed was kept constant at 7 m/min. The mice were exercised for 8 weeks. All mice when exercised tolerated the exercise protocol well throughout the study, and they did not show any signs of exhaustion. All the animal handling procedures were approved by the Institutional Animal Care and Use Committee of the UofL. The exercise protocol followed were in agreement with the American journal of veterinary research 24.

### Isolation of exosomes from heart and serum

The heart was digested using a protocol, previously described 25. The heart homogenate was sequentially filtered through a 40 μm mesh filter (BD, Franklin Lakes, NJ, USA) and a 0.2 μm syringe filter (Thermo Scientific, Rockford, IL, USA). The filtrate was passed through a 100 kD cut-off filter (Amicon ultra 15; MIlipore Tullagreen, Carrigtwohill, Co. Cork, Ireland). Exosomes were isolated from the concentrated filtrate by using ultracentrifugation. Briefly, the filtrate was sequentially centrifuged at 300 × g for 10 min. at 4°C, 2000 × g for 10 min. at 4°C, and at 10,000 × g for 30 min. at 4°C to discard cells, membranes and debris. The supernatant was centrifuged at 100,000 × g for 70 min. at 4°C to pellet exosomes. The exosome pellet was washed with cold PBS and resuspended in 1 ml of cold PBS (Invitrogen, Carlsbad, CA, USA).

### Confirmation of isolated exosomes (Electron microscopy, AChE activity)

Electron microscopy was done in the core imaging facility (Department of Anatomical Sciences and Neurobiology, University of Louisville) using the Phillips CM12 electron microscope with digital cameras. Briefly, the heart tissues were fixed in 3% glutaraldehyde and cut into ultrathin sections at 60–90 nm thick and collected onto grids. The grids were stained with uranyl acetate for negative staining. The exosomes were confirmed by AChE activity (based on the Ellman assay) 26. Briefly, 2 μl of exosomes were resuspended in PBS diluted in 298 μl of the AChE assay working solution [1.25 mM Acetylthiocholine (A5751; Sigma-Aldrich, St. Louis, MO, USA), 0.1 mM 5,5′-Dithio-bis(2-nitrobenzoic acid) (DTNB, D8130; Sigma- Aldrich) in 0.1 M phosphate buffer at pH 8.0] and incubated at 37°C in the dark for 30 min. Optical density (OD) was measured at 412 nm to quantify AChE activity in the exosome solution.

### Western blots

In addition, the exosomes were also identified by the expression of TSG101, CD81 and flottilin-1 antibodies with Western blots. The exosomes (20 μg) were separated by SDS-PAGE (20 μg) and transferred on to Hybond N+ by electrotransfer. The membranes were probed with primary antibodies (CD81, flottilin 1 and TSG101) followed by HRP conjugated secondary antibodies. The membranes were developed with ECL Western blotting detection system (GE Healthcare, Piscataway, NJ, USA) and the image was recorded in the gel documentation system ChemiDoc XRS system (Bio-Rad, Richmond, CA, USA). The data were analysed using Image Lab densitometry software (Bio-Rad) and normalized to TSG101.

### Flow cytometry

Since the exosomes are small, Dyna beads (Life Technologies, Grand Island, NY, USA) were used for flow cytometry according to the manufacturer’s protocol. Flow cytometry was perfomed for exosome quantification as described by our earlier report 5. Briefly, exosomes were incubated with magnetic beads (Dyna beads, Life Technologies) attached to the appropriate antibody (CD81/Flottilin1), in PBS at 4°C overnight with gentle agitation. The reaction was stopped by incubation with 100 mM glycine. Coated beads were washed thrice with PBS and labelled with anti CD81/Flottilin1 PE along with appropriate isotypic control. The exosomes were analysed by flow cytometry (BD Accuri™ C6 Flow Cytometer).

### Immunohistochemistry for colocalization of CD81 and flottilin 1

Heart tissues from the four group of mice were collected and embedded in disposable plastic tissue moulds (Polysciences Inc., Warrington, PA, USA) containing tissue freezing media (Triangle Biomedical Sciences, Durham, NC, USA). Cryosectioning was done on polylysine coated slides using Cryocut (Leica CM 1850, Buffalo Grove, IL, USA) to get tissue sections of 5 μm. Chilled methanol was used for tissue fixation and permeabilization was done by using Triton X 100 (0.3%). Primary antibodies were added for CD81 and flottilin 1 at 1:250 dilution (Abcam, Cambridge, MA, USA) and incubated overnight at 4°C. The slides were washed with TBS and TBST followed by 1 hr incubation with fluorescently tagged secondary antibodies. The slides were mounted and visualized with a laser scanning confocal microscope (Olympus FluoView1000, Pittsburgh, PA, USA).

### MicroRNA expression in exosomes

MicroRNAs were selected on the basis of sequence similarity with the 3′ region of the MMP9 gene as depicted by *targetscan* and *mirbase.org* websites. MicroRNAs were isolated from the exosomes using the Mirvana RNA isolation kit (Life Technologies) and amplified using the miscriptII Preamp PCR kit (Qiagen, Germantown, MD, USA). The microRNAs (466, 323-5p, 455, 29b) were quantified by qRTPCR using miscriptII RT kit (Qiagen) in the Stratagene Mx3000P real-time PCR machine and using the mcroRNA primer assays (Qiagen). The delta C_t_ method after normalizing the genes with snoRD-72 was used for the calculation of fold expression. For real time expression of MMP9 in heart tissue, RNA was isolated using the Trizol method and quantification was done by the Qiagen one step RTPCR kit.

### Activity of MMP9 2D zymography

To show active MMP9 in the tissue 2D zymography was used (a technique pioneered in our laboratory 19). Briefly, the tissue was minced in cocadylic acid and the tissue extract was mixed with ampholyte (pH 3–10; Invitrogen) to prepare the sample rehydration buffer as per manufacturer’s protocol. The zoom IPG strips (pH 3–10) were soaked in sample rehydration buffer in the zoom Immobilized PH Gradient runner cassette, overnight and isoelectrofocusing was done in the zoom IPG runner system (Invitrogen) using the step voltage program as per manufacturer’s instructions. The strip was placed on 10% SDS-PAGE gel prepared with 2% gelatin and electrophoresed until the dye passed out. The gel was washed three times in 2.5% Triton X-100 for 20 min. each to remove SDS and incubated in activation buffer (5 mmol/l Tris HCl- pH 7.4, 0.005% v/v Brij-35, and 1 mmol/l CaCl_2_) for 24 hrs at 37°C. The gel was stained in commasie and destained to observe a clear zone against blue background as a result of proteolytic acitivity of MMPs for gelatin, along with controls. The gels were imaged with gel documentation system (Bio-Rad, Hercules, CA, USA) and data were analysed using image lab software (Bio-Rad).

### Statistical analysis

The results were determined as mean ± SEM for each group. The difference between control and diabetic mouse, with and without exercise was determined by one-way ANOVA followed by pairwise comparison using Student’s *t*-test. Bonferroni correction was applied for low sample size. A *P*-value of less than 0.05 was considered significant.

## Results

### Exosomes confirmed by electron microscopy show Acetyl choline esterase activity

**Figure 1:**
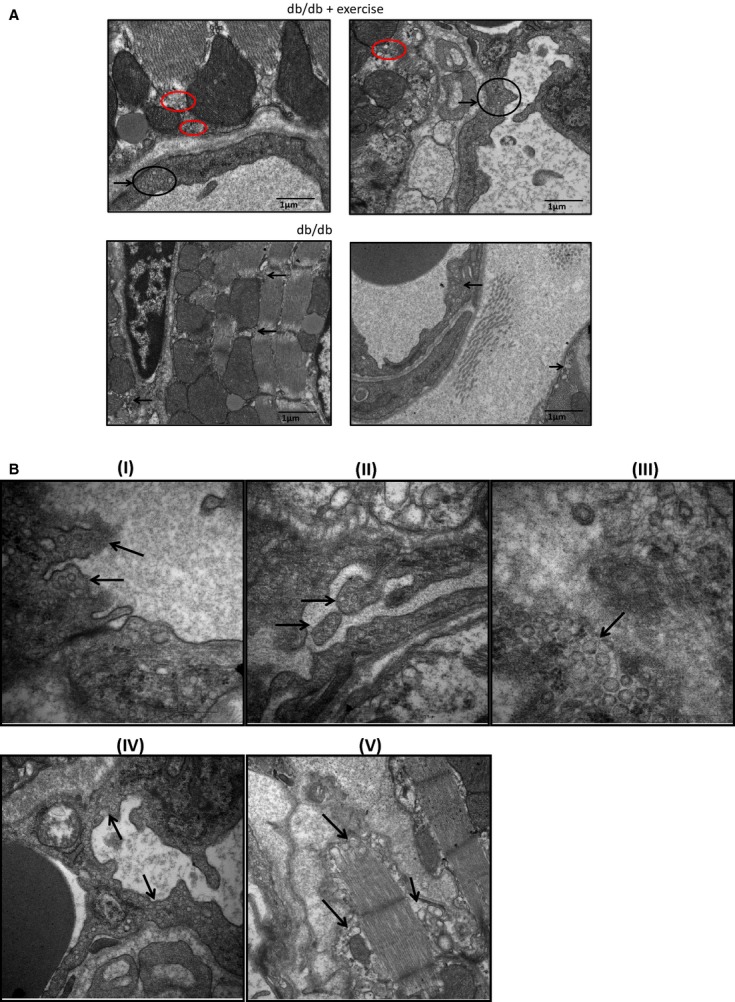
Electron microscopy of heart tissue. (A) Heart tissue from db/db exercise and non-exercise mouse were fixed in 3% glutaraldehyde and cut into ultrathin sections (60–90 nm thick) and collected onto grids. The grids were stained with uranyl acetate for negative staining. The exosomes were observed in the vessel wall (black circle) and in the close vicinity of myocytes and mitochondria (red circles). The exosomes were robust in the db/db exercise group. (B: I and II) Budding of the vessel wall to release the exosomes into the lumen (black arrows). (III) Exosomes in the extracellular space. (V) Exosomes in the wall of the vessel. (VI) Exosomes released by myocyte fibre.

**Figure 2:**
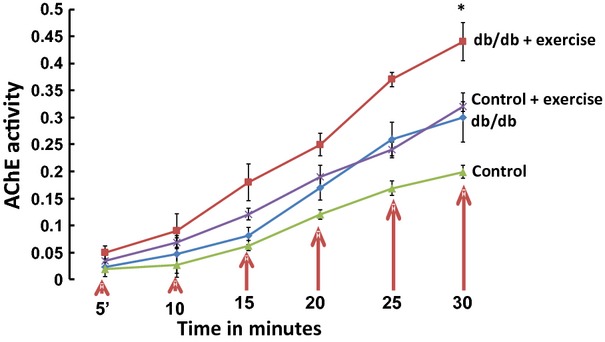
AChE activity in the exosomal fraction. The exosomes were isolated from the heart and serum of the exercise (db/db and control) and non-exercise (db/db and control) groups by ultracentrifugation. AChE activity was assessed in the exosomal fraction by Ellman assay 26. Different concentrations of exosomes were used to standardize the assay. The exosomes suspended in PBS were diluted in AChE working solution and incubated at 37°C in dark for 30 min. OD was measured at 412 nm at regular time intervals of 5 min. The exosomal fraction from the exercise group had significantly higher activity as compared to the non-exercise group (*P < 0.05).

The electron micrographs of the heart tissue confirmed the presence of exosomes in the extracellular space of myocytes and vessel walls (Fig.1). There was robust release of exosomes in the exercise group (db/db exercise) as compared to the controls (Fig.1A). We also observed the release of exosomes through budding (Fig.1B (I) & (II)) into the lumen of the vessel. The exosomes were evident in the extracellular space (Fig.1B(III)), in the vessel walls (Fig.1B(IV)) and in the close vicinity of myocytes (Fig.1B(V)). The exosomes isolated from the serum samples were further confirmed by acetylcholine esterase activity (Fig.2). The AChE activity was higher in the exercise group.

### Exosomes contain markers CD81 and Flottilin 1

**Figure 3:**
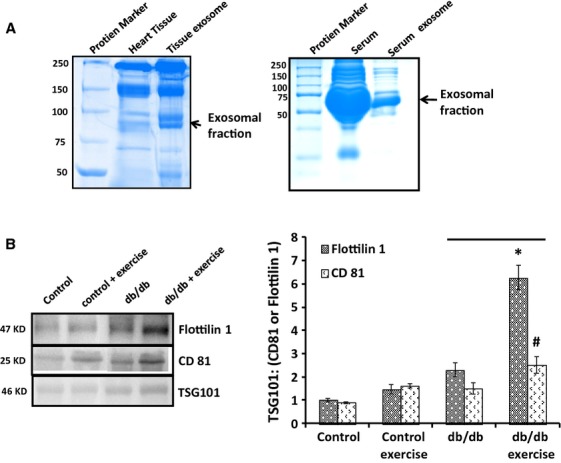
Confirmation of exosomal markers CD81 and flottilin 1. The exosomal fraction from both heart and serum was separated on SDS PAGE (A) and transferred to hybond N^+^ for Western blotting. The first lane shows the exosomes from the serum of db/db control mice (Jackson Laboratory) without exercise while the second lane shows the same with exercise. (B) The exosomal fractions showed higher expression of CD81 and flottilin 1 proteins in the exercise group as compared to the non-exercise group.

**Figure 4:**
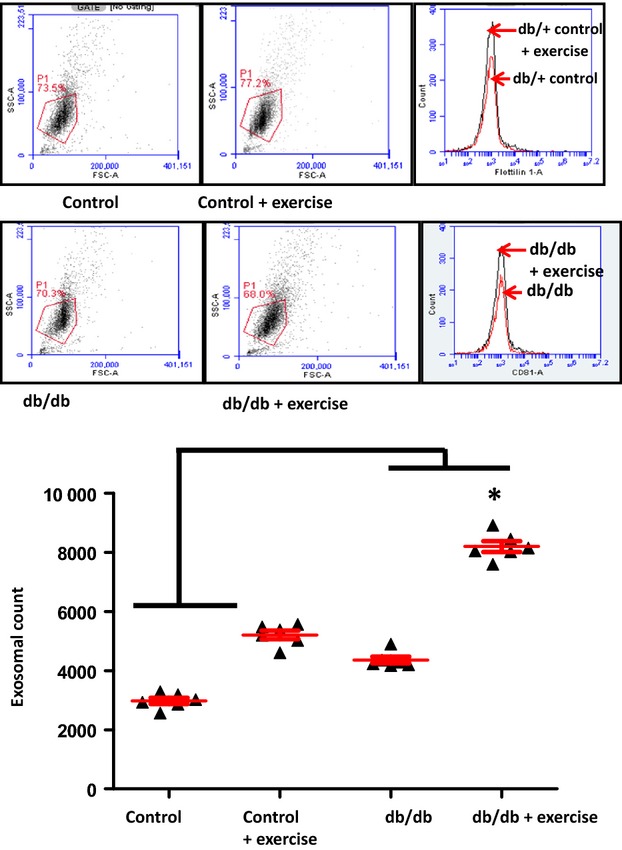
Quantitative estimation of exosomes by flow cytometry. The exosomes were quantitated by using Dyna beads from Life Technologies according to the manufacturer’s protocol. The Dyna beads were incubated with the primary antibodies followed by incubation with the exosomal fraction. The Dyna beads containing the exosomes were subjected to flow cytometry and we observed that there was significant increase in the number of exosomes in the exercise group as compared to the non-exercise (*P < 0.05), *n* = 6.

The exosomes isolated from serum and tissue were analysed by SDS-PAGE (Fig.3A) and Western blotting (Fig.3B) for the expression of markers CD81 and flottilin 1. In db/db+exercise group, there was enhanced expression of the CD81 and flottilin 1 markers (Fig.3B) as compared to db/db non-exercise. Similarly, the control group with exercise expressed higher levels of both the markers as compared to control non-exercise group (Fig.3B). The quantitative estimation of exosomes was done by flow cytometry for the exercise and the non-exercise group using CD81 and flottilin 1 antibodies. Flow cytometry revealed greater population of exosomes in the db/db exercise group as compared to db/db non-exercise group (Fig.4).

### Colocalization of CD81 and flottilin 1 in the tissues

**Figure 5:**
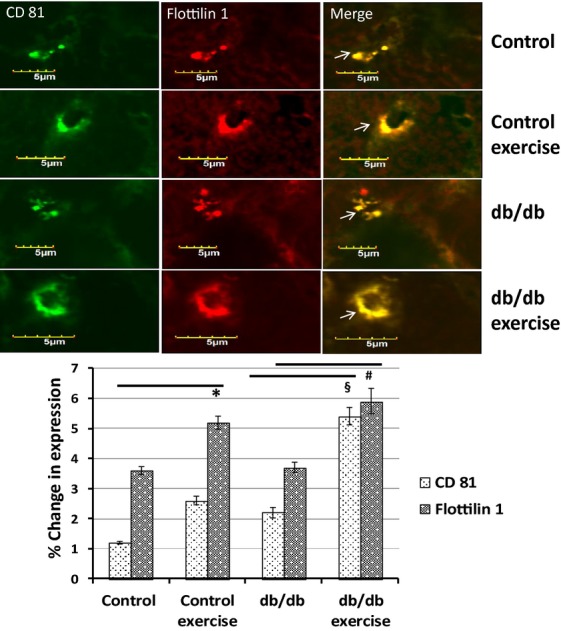
Colocalization of CD81 and flottilin 1 in heart tissue. Immunohistochemistry of the cryosectioned tissues with CD81 and flottilin 1 antibodies showed their colocalization in the heart tissue. We observed in electron microscopy that exosomes were concentrated in the vessel walls. We observed the same pattern of colocalization of CD81 and flottilin 1 in the vessel wall in immunohistostaining. There was enhanced colocalization of both the markers in the exercise group as compared to non-exercise (*P < 0.05).

Immunohistostaining of the heart tissues showed that there was colocalization of CD81 and flottilin 1 in the heart tissues (Fig.5). In the electron microscopy, we observed that exosomes were concentrated in the vessel walls. We found the same pattern of colocalization in the vessel walls of heart. The colocalization of CD81 and flottilin 1 was higher in the exercise group as compared to the non-exercise.

### Enhanced expression of microRNAs (29b and 455)

**Figure 6:**
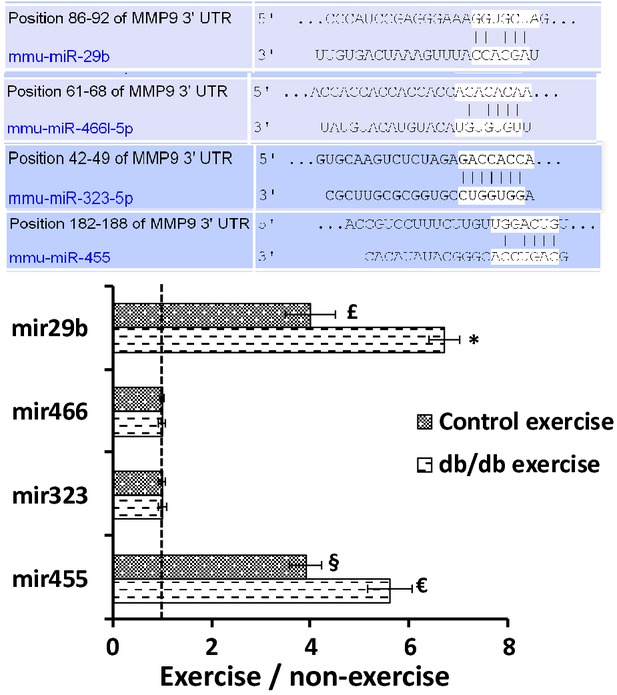
Expression of mir29b, mir455, mir323-5p and mir466. microRNAs were isolated from the exosomal fraction and quantitated by qRT-PCR. We selected these microRNAs based on their highest sequence similarity (>70%) to the 3′ region of the MMP9 gene. Of the four microRNAs there was significant upregulation of mir29b and mir455 (*P < 0.05) in the exercise group.

**Figure 7:**
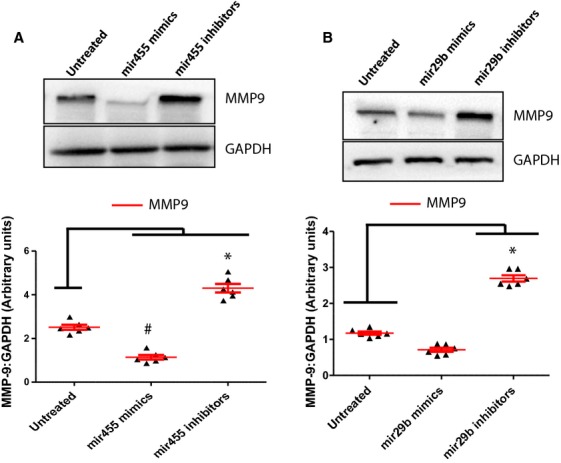
Use of mimics and inhibitors to evaluate the regulation of MMP9. We used mimics and inhibitors for mir455 and mir29b in the HL-1 cell line (as described in our previous study 27) to evaluate the expression of MMP9. We observed that mir455 tightly regulated the expression of MMP9 since the use of mir455 mimics diminished the expression of MMP9 (A). With mir29b the use of mimics did not completely downregulate the expression of MMP9 (B). The data suggest that mir29b may not regulate MMP9 directly.

We evaluated the expression levels of mir323-5p, 455, 466 and 29b by qRT-PCR. These microRNAs showed highest sequence similarity (>70%) to the 3′ region of MMP9 as depicted by *targetscan* and *mirbase.org* (Fig.6). Although qRT-PCR confirmed the presence of all the microRNAs in the exosomes, the expression of mir29b and mir455 was significantly upregulated in the exercise group as compared to the non-exercise group. As these microRNA can bind to MMP9 and downregulate its expression, we checked the expression of MMP9 by RT-PCR, qRT-PCR and IHC. We used mimics and inhibitors in HL-1 cell line (as reported earlier 27) to evaluate whether these microRNAs regulate the expression of MMP9. We observed that mir455 mimics downregulated the expression of MMP9 while mir455 inhibitors upregulated the expression (Fig.7A). We observed the same trend with mir29b but it was not significant. Mir29b mimics did not completely inhibit the expression of MMP9 suggesting that it may not regulate MMP9 directly and there may be other mechanisms (Fig.7B).

### Exercise downregulated MMP9

**Figure 8:**
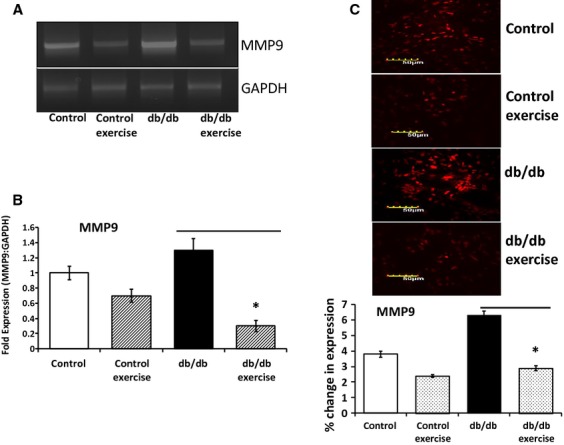
MMP9 expression after exercise. Downregulated expression of the activated MMP9 was observed after exercise in the db/db mouse as compared to the control mouse. The MMP9 transcript was evaluated by RTPCR and qRTPCR (A and B). Immunohistostaining also confirmed downregulated expression of MMP9 in the exercise group as compared to the non-exercise group (C).

**Figure 9:**
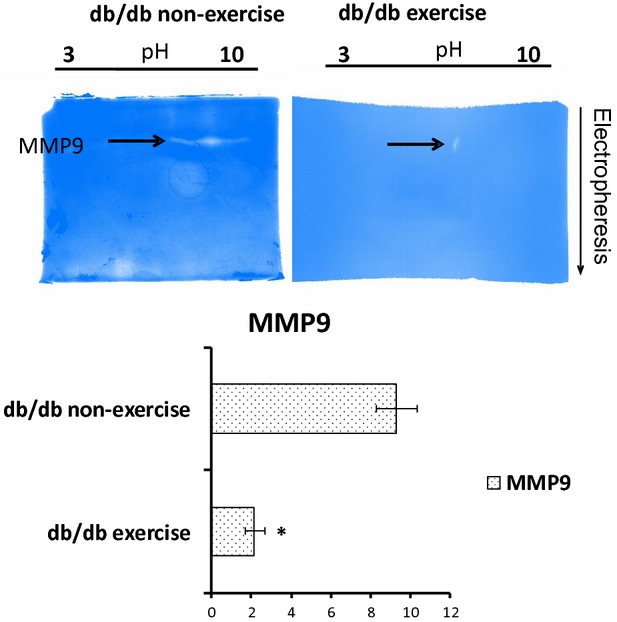
MMP9 activity by 2D zymography. The activity of MMP9 was assessed by 2D zymography (a technique pioneered in the PI’s laboratory 19). The samples were mixed with ampholyte (pH 3–10) and soaked with IPG strips (pH 3–10). After performing Isoelectric focusing the strips were loaded in the second dimension on to PAGE gels containing 2% gelatin. The gel was washed with Triton X100 and incubated with developing buffer for 48 hrs. We observed that in the exercise group, there was significant reduction in the activity of the MMP9 which suggests that exercise can prevent the deleterious effects of MMP9 that lead to fibrosis and uncoupling.

The expression of MMP9 was downregulated in the heart tissue after exercise as evaluated by RT-PCR and qRT-PCR (Fig.8A and B). The immunohistostaining of the heart tissue also confirmed the downregulated expression of MMP9 in db/db mouse with exercise, though there was not significant change in the MMP9 expression in the control group with and without exercise (Fig.7C). The activity of MMP9 was assessed by 2D zymography and we observed decrease in the activity of MMP9 in the 2D zymography gels after exercise (Fig.9).

## Discussion

Our study strongly demostrated that in exercise exosomes are released that downregulate the levels of MMP9 by means of microRNAs 29b and 455 and prevent the downstream detrimental effects of MMP9 that lead to fibrosis and myocyte uncoupling. The electron microscopy of the heart tissues revealed the accumulation of exosomes in the vessel walls which was further confirmed by the colocalization of CD81 and flottilin 1 in the heart vessels by immunohistostaining. The exosomes were seen to be released by evagination of the vessel lumen wall into the blood stream by electron microscopy. Hence, in part this study explains the molecular aspects of exercise benefits in the heart of diabetic patients.

The presence of exosomes in the cardiosphere which can be termed as ‘cardiosomes’ is a recent finding, where they are believed to carry microRNAs and other paracrine factors that can benefit the surrounding cells 18,28,29. We have reported earlier the role of exosomes in endothelial dysfunction 5. Exosomes which are derived from human plasma have been shown to contain RNAs that are characterized by deep sequencing 30–32. Sahoo and Losordo (2014) and Waldenstrom and Ronquist (2014) have reviewed the role of exosomes in cardiac remodelling by acting as a second messenger between progenitor cells and cardiomyocytes 11,12. The electron microscopy images showed by these two studies demonstrate that exosomes are present in multivesicular bodies, near myocytes and endothelial cells. Exosomes have also been reported as critical agents of cardiac regeneration triggered by cell therapy 10. Our current study also shows release of exosomes in the cardiosphere which is in agreement with these studies, however, the role of exosomes have not been shown in exercise. Exercise in diabetic patients mitigates cardiovascular complications and has been described as a polypill 1, although, the molecular mechanisms explaining the cardiac benefits of exercise in diabetic patients are unexplored. Wang *et al*. (2014) have reported that insufficient myocardial angiogenesis in type 2 diabetic rats is mediated by exosomes that carry mir320, and transfer it to the endothelial cells 17. Interestingly, we observed mir29b and mir455 in the exosomal population after exercise in type 2 diabetic mice which suggests that the exosomal content varies and depends upon the cells of origin at time of secretion.

Although there are no reports of exosomes in exercise, there is some indication that skeletal muscle function may involve circulating microRNAs encapsidated in exosomes 16. Another report by Aswad *et al*. (2014) suggests that in lipid induced insulin resistance mice, the muscle homeostasis is affected by exosomes 33. Hence, our findings on exosome mediated benefits of exercise in diabetic patients are novel. We also observed that the activated MMP9 is downregulated after exercise in muscles 34,35. A study by Shon *et al*. (2011) demonstrated that exercise attenuates MMP activity in the atherosclerotic plaques in ApoE mice 2. Activated levels of MMP9 decreases cardiac tensile strength 20 and are crucial mediators of cardiac remodelling 19,36,37. High levels of glucose in diabetes upregulates the expression of MMP9 and MMP13 in tendon cells 38 and the elevated MMP9 levels can further lead to cardiac fibrosis and myocyte uncoupling. Since exercise brings down the levels of MMP9 it can prevent fibrosis and uncoupling in diabetic patients. We found that downregulation of MMP9 was associated with upregulation in the levels of mir29b and mir455 in the exosomes. These microRNAs show high sequence similarity to the 3′ region of MMP9 and hence can regulate MMP9 after exercise. The use of mimics and inhibitors in the HL-1 cell line 27 confirmed that mir455 directly regulated MMP9. However, with mir29b the results were not significant and suggested that there may be other mechanisms that are involved in the regulation of MMP9 by mir29b 39. The evidence provided by this study that exosomes released in exercise contain microRNAs (mir29b, mir455) to silence MMP9 can be extrapolated to replace MMP9 inhibitors which have not been successful so far for managing cardiac remodelling 23.
